# Evidence for the circulating microRNA hsa-let-7d-3p as a potential new biomarker for sepsis in human subjects

**DOI:** 10.1186/s40001-022-00763-3

**Published:** 2022-07-30

**Authors:** Zhaorui Zhang, Hailun Luo, Chunsun Li, Zhixin Liang

**Affiliations:** 1 Present Address: Department of Pulmonary and Critical Care Medicine, The Eighth Medical Center of Chinese, PLA General Hospital, No. 28 Fuxing Road, 100853 Beijing, China; 2 Department of Respiration, LiangXiang Hospital, Fangshan, Beijing, China

**Keywords:** Sepsis, Biomarkers, Diagnosis, Hsa-let-7d-3p, miRNAs

## Abstract

**Background:**

Current biomarkers for the early detection of sepsis have low sensitivity and specificity. Serum microRNAs (miRNAs) have been proposed as novel noninvasive biomarkers for various diseases. The aim of the present study was to discover a novel diagnostic biomarker for sepsis in human subjects.

**Methods:**

miRNA expression profiling was performed using peripheral blood from three sepsis patients in the sepsis stage and improved condition stage using microarray screening. The differentially expressed miRNAs were primary validated by real-time quantitative polymerase chain reaction (RT-qPCR) in a further set of 20 sepsis patients in the sepsis stage and improved condition stage. Finally, we validated the differentially expressed miRNAs in 95 sepsis patients and 66 nonsepsis patients. The validated miRNAs and patients’ clinical indictors were analysed in a multivariate logistic regression model. The diagnostic value of the changed miRNA in sepsis was determined and compared with CRP and WBC by analysing the receiver operating characteristic (ROC) curves.

**Results:**

According to the criteria, we detected 11 miRNAs regulated by the miRNA chip. RT-qPCR detection showed that the expression of hsa-let-7d-3p in sepsis patients was upregulated compared with that in nonsepsis patients. In a multiple logistic regression analysis, serum miRNA hsa-let-7d-3p was found to be an independent predictor of sepsis. Receiver operating characteristic curve (ROC) analysis showed that the area under the ROC curve of serum hsa-let-7d-3p was 0.696 [95% confidence interval (0.615, 0.778)].

**Conclusion:**

The miRNA hsa-let-7d-3p was identified as a novel biomarker for the early detection of sepsis.

**Supplementary Information:**

The online version contains supplementary material available at 10.1186/s40001-022-00763-3.

## Background

Sepsis should be defined as life-threatening organ dysfunction caused by a dysregulated host response to infection. Despite the development of diagnostic and therapeutic techniques, sepsis is still the major cause of mortality in critically ill patients in the past 20 years [1, 2]. Approximately 1/5–1/2 of patients with sepsis die of multiorgan dysfunction syndrome [3, 4]. Delayed diagnosis or accurate assessment of patients with sepsis usually increases the heavy economic burden and mortality rate [5–7]. Current biomarkers, such as C-reactive protein (CRP) and procalcitonin (PCT), have been widely applied in the clinic to monitor sepsis. However, a meta-analysis showed CRP had low sensitivity and specificity to indicate an infection and thus it is not recommended in the current sepsis guidelines as a sepsis biomarker [8]. PCT is better than CRP in the diagnosis of bacterial infection but still lacks specificity [9, 10]. Therefore, biomarkers that can be used to establish a diagnosis for sepsis are still not well established [7].

miRNAs are a class of 18–25 nucleotide noncoding RNAs that regulate gene expression at the posttranscriptional level and play a role in diverse biomolecular processes, including inflammation and immunity [11, 12]. Studies have shown that miRNAs are present in a cell-free form (serum, plasma or other body fluids), and the levels of individual miRNAs or specific miRNA signatures are linked to various cancers or other diseases [13–15]. Recently, previous studies reported that miR-25 and miR-122 may be related to sepsis; however, the diagnostic values of these miRNAs were unsatisfactory [8]. Novel biomarkers that can accurately guide the diagnosis and treatment of sepsis are still needed.

In this study, we hypothesized that sepsis-related miRNAs in serum have the potential to be diagnostic biomarkers. Accordingly, the goal of the present study was to discover miRNAs that can be used to diagnose sepsis early in critically ill patients.

## Methods

### Ethics

This study was approved by the ethics committee of the General Hospital of PLA, No. 2014-048-01. All subjects or their agents signed informed consent forms.

### Patients and sepsis definition

The study population consisted of 161 patients, 95 sepsis patients and 66 nonsepsis patients, from April 2012 to May 2014. Patients newly hospitalized in the ICU of the PLA Hospital were screened as candidates. Patients were included if they met both of the following criteria: (1) the patient had an infection, and (2) Sequential Organ Failure Assessment (SOFA) ≥ 2 [16]. Patients were excluded according to the following criteria: (1) age < 18 years; (2) pregnancy or lactation; (3) HIV infection; and (4) unwillingness to participate. Patients who died or were discharged within 24 h after admission to the ICU were excluded from the study. According to the third International Consensus Definitions for Sepsis [16], patients were independently classified as having no sepsis or sepsis at the time of admission by two intensivists. Agreement concerning the diagnosis was achieved in all cases. In clinical practice, antimicrobial therapy was prescribed according to the usual practice of the ICU when infection was suspected by the attending physicians, without interference by this study. The age, sex, and Sequential Organ Failure Assessment (SOFA) scores were recorded, and the levels of C-reactive protein (CRP) and procalcitonin (PCT) in serum were analysed routinely. The condition improvement of sepsis patients was defined as follows: (1) the patient was diagnosed with infection; (2) Sequential Organ Failure Assessment (SOFA) ≥ 2. Three patients with improved sepsis were selected for miRNA screening, and 20 patients with improved conditions were selected for primary validation.

### RNA isolation and quality assessment

Total RNA from peripheral blood was isolated and purified with a TRI Reagent BD kit according to the manufacturer’s instructions. RNA quality and quantity were measured by using a Nanodrop spectrophotometer (ND-1000, Nanodrop Technologies). RNA integrity was assessed by agarose gel electrophoresis.

### miRNA gene chip analysis

The sera of 3 sepsis patients in the sepsis stage and improved condition stage were collected. The samples underwent microarray analysis using the 7th generation of the miRCURY LNA^™^ miRNA Array (Exiqon A/S, Vedbaek, Denmark). Genes with Benjamini–Hochberg false discovery rate (FDR)-corrected p values of 0.05 and threshold values of fold change  ≥ 2 or < 0.5 were considered to be differentially expressed.

### Real-time quantitative polymerase chain reaction

The serum of 20 sepsis patients in the sepsis stage and improved condition stage were collected. Primary RT-qPCR analysis was conducted to detect the expression level of the selected miRNAs in the patients with sepsis for validation.

A final RT-qPCR analysis was conducted to detect the expression levels of the primary validated target miRNAs in patients with sepsis and patients without sepsis.

Total RNA was reverse transcribed to cDNA using a PrimeScript^™^ RT Reagent Kit with gDNA Eraser according to the manufacturer’s instructions (TIANScript RT Kit, Beijing, China). Twenty-microlitre volume reactions were used in real-time quantitative polymerase chain reaction (RT-qPCR) containing 4 μl RNA (50 μl purified serum), 2 μl Bulge-Loop^™^ RT primer (500 nM) (RiboBio, Guangzhou, China), 2 μl Super Pure dNTPs, 6.5 μl nuclease-free H_2_O, 4 μl 5 × First-Strand Buffer, 0.5 μl RNasin, and 1 μl TIANScript M-MLV. The reaction setting consisted of a predenaturation step for 1 min at 95 °C followed by 40 cycles of denaturation for 10 s at 95 °C, 20 s at 60 °C and 10 s at 70 °C. The samples were heated for 1 min at 95 °C, 1 min at 60 °C, and then heated from 55 °C to 100 °C for the melt curve. The expression level of each miRNA was analysed by the comparative Cq method (2^−△Cq^). cel-miR-39-3p was used as a reference gene as previously described [17].

### Statistical analysis

Data were statistically analysed using SPSS 17.0. In the univariate analysis, data with normal and nonnormal distributions and count data were evaluated through *t* tests, Mann–Whitney tests, and *χ*^*2*^ tests, respectively. *p* < 0.05 was considered a significant difference. A multivariable logistic regression analysis was also conducted to evaluate the predictive values and odds ratios of the miRNAs. The specificity and sensitivity of miRNAs in the diagnosis of sepsis were calculated in terms of the area under the receiver operating characteristic (ROC) curve.

## Results

### Clinical characteristics of the subjects

The clinical characteristics of the patients are shown in Table 1. The study enrolled 161 patients with 95 patients with sepsis and 66 without sepsis, including 105 males (65.2%) and 56 females (34.8%) with an average age of 64.0 (47.5–78.0) years old. Approximately 95 patients with sepsis, including 68 males (71.6%) and 27 females (28.4%), with an average age of 67.0 (53.0–81.0) years old, were diagnosed by sepsis criteria. The comorbidities and platelets between sepsis patients and nonsepsis patients were comparable, without significant differences. As anticipated, sepsis patients had higher SOFA scores, APACHE II scores, WBC counts and mortality rates (*p* < 0.05). The details of 3 patients included in the miRNA screening and 20 patients in the primary validation are shown in the supplemental materials.

**Table 1:** Clinical characteristic of patients with sepsis and no sepsis

Characteristic	All patients (*n* = 161)	Nonsepsis (*n* = 66)	Sepsis (*n* = 95)	*p* value
Gender (male), *n* (%)	105(65.2)	37(56.1)	68(71.6)	< 0.05
Age, (years) Female	64.0(47.5–78.0)	53.5(43.0–71.25)	67.0(53.0–81.0)	< 0.05
SOFA score	5.0(3.0–7.5)	3(2–5)	5(4–9)	< 0.05
APACHEII score	14.0(10.0–20.0)	11.0(9.0–15.0)	16.0(12.0–22.0)	< 0.05
WBC counts(× 10^9^/L)	10.4(7.4–14.7)	9.2(6.9–12.6)	11.7(7.7–16.8)	< 0.05
Platelet counts(× 10^9^/L)	150.0(84.3–222.3)	148.0(107.0–220.5)	154.0(78.0–224.0)	NS
CRP (mg/dl)	7.81(3.6–13.1)	4.3(1.0–9.1)	10.8(5.6–15.3)	< 0.05
Comorbidity conditions, *n* (%)				
COPD	21(13.0)	10(15.2)	11(11.6)	NS
Heart failure^a^	17(10.6)	7(10.6)	10(10.5)	NS
Coronary heart disease	37(23.0)	14(21.2)	23(24.2)	NS
Hypertension	59(36.6)	21(31.8)	38(40.0)	NS
Cerebrovascular disease	24(14.9)	7(10.6)	17(17.9)	NS
Diabetes mellitus	40(24.8)	17(25.8)	23(24.2)	NS
Immunosuppression	10(6.2)	1(1.5)	9(9.5)	NS
Cancer	13(8.1)	8(12.1)	5(5.3)	NS
Acute pancreatitis	7(4.3)	5(7.6)	2(2.1)	NS
Mortality^b^, *n* (%)	42(26.1)	6(9.1)	36(37.9)	< 0.00005

*SOFA* Sequential Organ Failure Assessment; *APACHE II* Acute Physiology and Chronic Health Evaluation II; *NS* no significance; *COPD* chronic obstructive pulmonary disease

^a^New York Heart Association class III–IV

^b^Patients in hospital more than 60 days was considered alive

### Overview of differentially expressed miRNAs in sepsis

Eleven miRNAs, namely, hsa-miR-373-5p, hsa-miR-495-3p, hsa-miR-642a-3p, hsa-miR-5584-3p, hsa-miR-2682-3p, hsa-miR-381-5p, hsa-miR-3591-5p, hsa-miR-3685, hsa-miR-501-5p, hsa-miR-4795-3p, and hsa-let-7d-3p, satisfied the screening criteria of *p* < 0.05 and a fold change ≥ 2 or ≤ 0.5. The fold changes of the miRNAs are shown in Table 2.

**Table 2:** Differentially expressed miRNAs in gene chip analysis

miRNA	Fold change
hsa-miR-373-5p	3.248689
hsa-miR-495-3p	2.86105
hsa-miR-642a-3p	4.738026
hsa-miR-5584-3p	0.220295
hsa-miR-2682-3p	0.144371
hsa-miR-381-5p	0.179186
hsa-miR-3591-5p	0.136297
hsa-miR-3685	0.147137
hsa-miR-501-5p	0.110752
hsa-miR-4795-3p	0.108122
hsa-let-7d-3p	0.194955

### Primary miRNA confirmation and selection by RT-qPCR

**Fig. 1:**
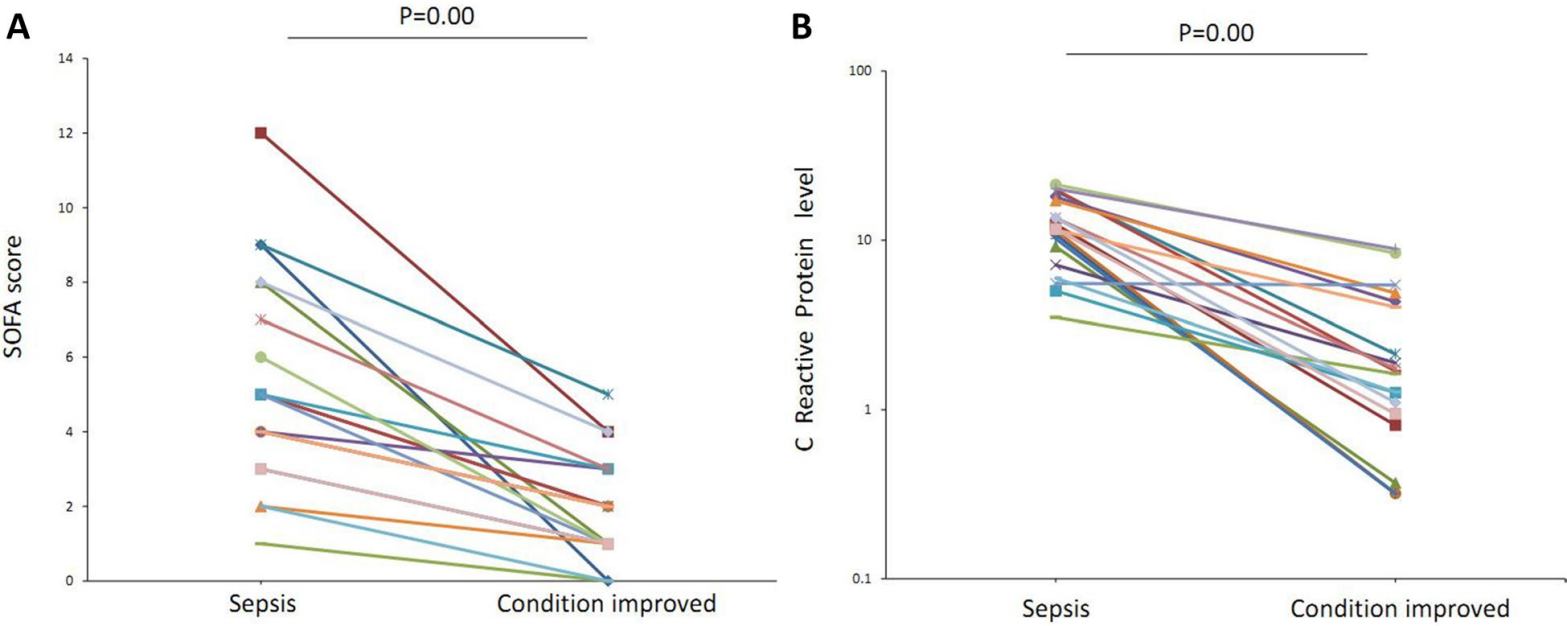
SOFA scores and CRP levels in sepsis patients in the sepsis stage and improved condition stage. **A** SOFA scores; **B** C-reactive protein levels (mg/L)

**Fig. 2:**
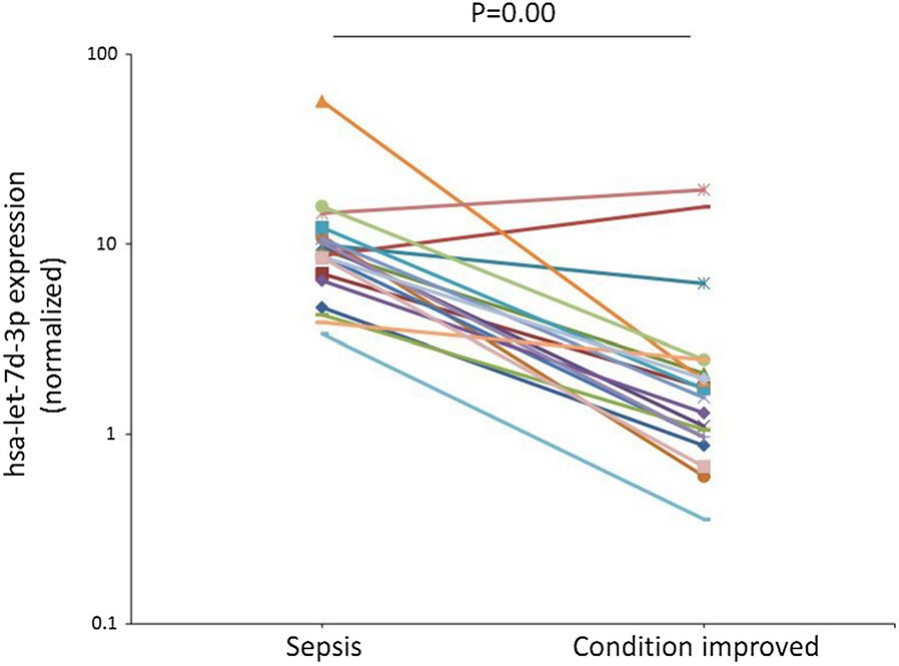
hsa-let-7d-3p expression in sepsis patients in the sepsis stage and improved condition stage

The relative data regarding miRNAs in sepsis were reviewed, and we selected hsa-miR-3591-5p, hsa-miR-501-5p, and hsa-let-7d-3p for primary validation. The serum of the patients was collected in the sepsis stage and condition improvement stage. The interval between the serum collection was from 6 to 37 days. The median SOFA scores and CRP levels were significantly reduced when the condition of the patients improved (Fig. 1). The results of RT-qPCR also indicate that the expression levels of hsa-let-7d-3p were significantly increased when the condition of the patients improved (Fig. 2). The fold changes of the selected miRNAs are shown in Table 3. The expression of hsa-let-7d-3p was significantly higher than that in the control group (*p* < 0.05). The 95% confidence interval was 6.49 (4.33, 9.22). The expression of the other two miRNAs was lower than that in the control, but the difference was not significant (*p* > 0.05).

**Table 3:** Fold change of the miRNA by self-control comparison in 20 patients

microRNA	Fold change(95% CI)	*p*-value
hsa-miR-3591-5p	0.72(0.42, 1.13)	0.18
hsa-miR-501-5p	0.68(0.44, 1.02)	0.08
hsa-let-7d-3p	6.49(4.33, 9.22)	0.00

### Final validation of hsa-let-7d-3p detected by RT-qPCR

**Fig. 3:**
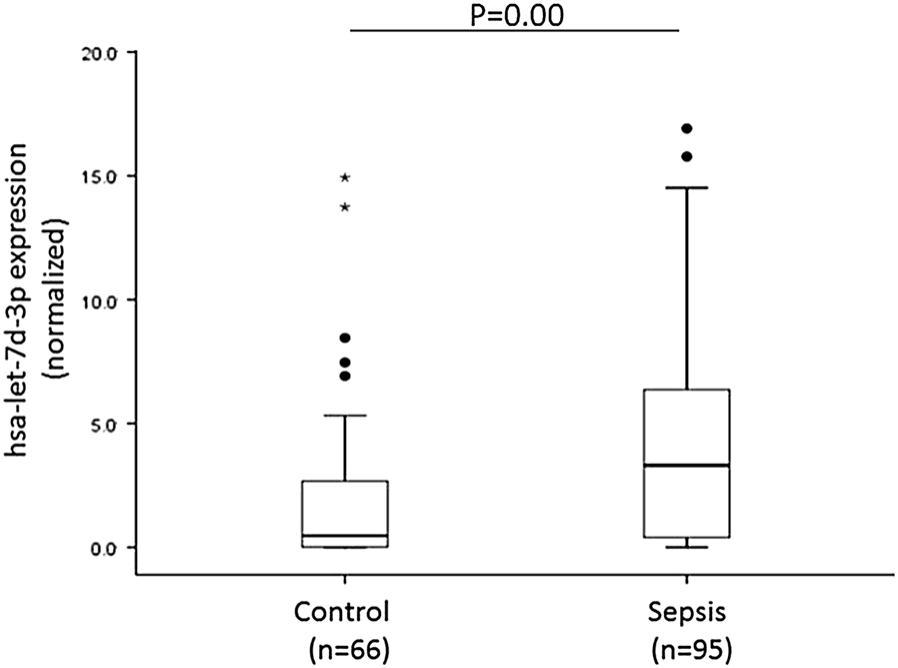
ΔCq value of hsa-let-7d-3p in the sepsis and control groups

In this step, 161 patients, including 95 sepsis patients and 66 nonsepsis patients, were included in the final RT-qPCR validation. The relative expression levels of hsa-let-7d-3p in sepsis patients were significantly higher than those in nonsepsis patients (*p* < 0.01) (Fig. 3). In addition, the altered miRNA expression patterns in the validation set were consistent with the results from the primary validation set.

### Multivariable logistic regression analysis

Furthermore, a logistic regression model was used to evaluate variables associations such as hsa-let-7d-3p along with age, SOFA score, WBC counts, CRP and trauma (Table 4). In the analysis, only the hsa-let-7d-3p and SOFA score showed statistically significant differences (*p* < 0.05). The results indicate that hsa-let-7d-3p and the SOFA score were independent predictors for the diagnosis of sepsis.

**Table 4:** Multivariate logistic regression analyses of hsa-let-7d-3p and the clinical indicators between sepsis patients and nonsepsis patient

Variable	Coefficient	Standard error	Wald	*p* value	Odds ratio
Age	0.025	0.011	5.320	0.021	1.026
SOFA score	0.217	0.068	10.170	0.001	1.242
WBC counts	0.069	0.039	3.021	0.082	1.071
CRP	0.059	0.032	3.424	0.064	1.061
hsa-let-7d-3p	0.143	0.062	5.336	0.021	1.153
Trauma	−1.107	0.614	3.254	0.071	0.331

### Comparisons of the diagnostic values of the miRNA, CRP and WBC counts

**Fig. 4:**
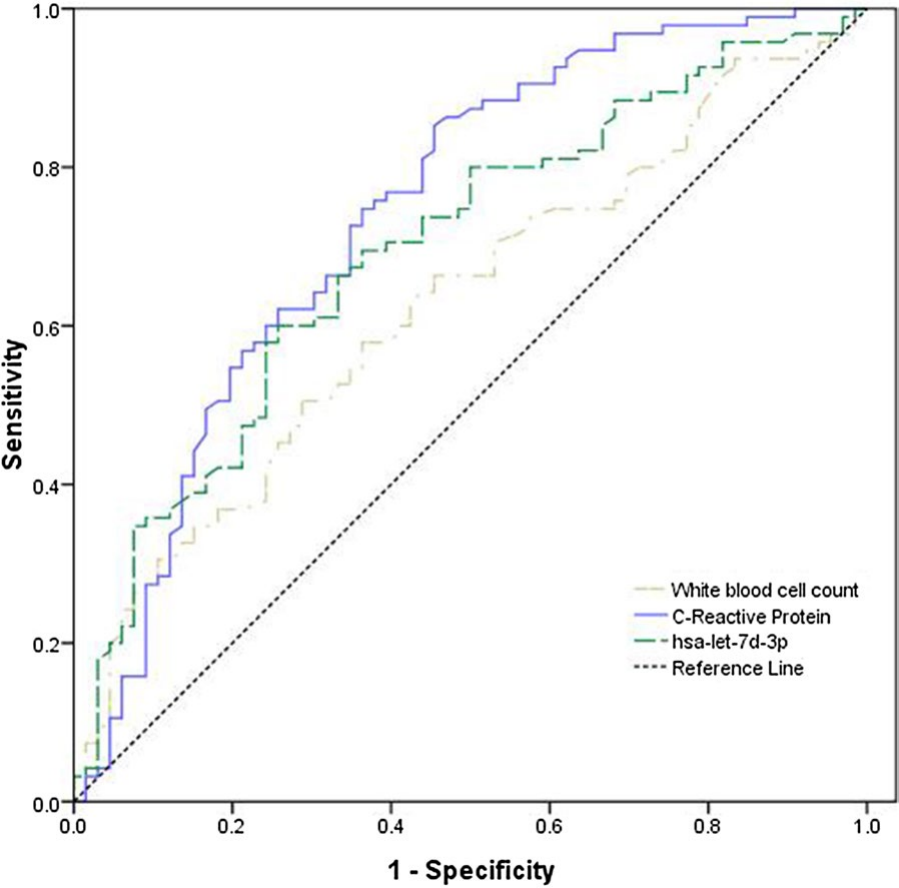
ROC curves of hsa-let-3p-d, CRP and the WBC count. The green dotted line represents hsa-letp-7d-3p; the purple solid line represents C-reactive protein; the white dotted line represents the white blood cell counts

The predictive capability of three variables (CRP, hsa-let-7d-3p, and the WBC count) was analysed using the ROC curves. ROC analyses of the biomarkers to predict sepsis showed that the areas under the curve were 0.742 (95% CI 0.662–0.822) for CRP, 0.696 (95% CI 0.615–0.778) for hsa-let-7d-3p, and 0.627 (95% CI 0.541–0.713) for the WBC count (Fig. 4).

The results suggest that serum hsa-let-7d-3p levels can be used to diagnose sepsis early. The best cut-off value of hsa-let-7d-3p for sepsis diagnosis was 2.6, with a sensitivity of 60% and a specificity of 75%.

## Discussion

miRNAs have been used as biomarkers for various types of disease since the discovery of circulating miRNAs in human peripheral sera. Sepsis represents a dysregulated immune response to infection that leads to organ dysfunction. A wide range of biomarkers are being investigated for the early identification of sepsis. These biomarkers include the measurement of acute phase proteins, cytokines, chemokines, damage-associated molecular patterns, endothelial cell markers, leukocyte surface markers, nonencoding RNAs (miRNAs, lncRNAs, and circulating RNAs) and soluble receptors, as well as metabolites and alterations in gene expression (transcriptomics) [17]. Traditionally, protein biomarkers such as C-reactive protein, procalcitonin, angiopoietins, and serum lactate have been used as biomarkers for sepsis. However, because miRNAs inhibit the translation of target mRNA into proteins, earlier detection of pathology may be identified with changes in miRNA expression [18]. Therefore, we examined microRNAs as a biomarker for sepsis. Our study demonstrates that hsa-let-7d-3p has the potential to be a novel diagnostic biomarker of sepsis, with pretty good sensitivity and specificity. Previous studies have found several miRNAs to be diagnostic and prognostic biomarkers for sepsis [8]. Yao and colleagues found that miR-25 displayed a superior diagnostic accuracy for sepsis compared to well-established markers such as CRP and PCT according to a ROC curve analysis in a well-characterized cohort of 70 patients with sepsis and 30 patients with noninfectious SIRS [19]. They further found that decreased miRNA-25 levels were related to the level of oxidative stress indicators in sepsis patients. Xie et al. found that circulating miR-122 was a potential biomarker of critical illness and sepsis in a sample of 214 patients with sepsis (117 survivors and 97 nonsurvivors), and Mir-122 predicted patients’ short- and long-term survival with high accuracy [20, 21]. hsa-let-7d-3p is one of the tumour suppressive let-7d family members. Let-7d is downregulated in numerous types of cancer, including ovarian cancer, and directly targets various oncogenes [22]. Gunal reported that hsa-let-7d-3p could downregulate the HMGA2 and KRAS genes and that the loss of hsa-let-7d-3p expression led to the progression of epithelial ovarian cancer related to tumorigenesis, invasion, and metastasis [22].

In our study, gene chip analysis revealed that 11 miRNAs satisfied the screening criteria of *p* < 0.05 and fold change ≥2 or <0.5. Limited data have been presented regarding the relationship of these 11 miRNAs with sepsis because of the following factors: (1) sepsis is a complicated syndrome with multiorgan failure in pathogenic microorganisms, severity and comorbidities. The complexity of sepsis leads to the complexity of sepsis biomarkers, and it is difficult to find a single biomarker of sepsis with high sensitivity and specificity. The miRNAs that can be used for the early diagnosis of sepsis vary in different studies.

In our study, the number of samples in the gene chip analysis was limited to the serum of 3 patients, but we further verified the results of the gene chip investigation through RT-qPCR analysis to compensate for the limitations of our study.

There are several advantages of our study. First, we chose a self-control comparison to primarily validate the differentially expressed miRNAs between the sepsis stage and improved condition stage. Self-control studies can maximally reduce influencing factors such as age, sex, illness severity, and comorbidities. Therefore, the results of our study are more convincing than those of other studies. Second, the cel-mirRNA-39-3p gene was chosen as a reference gene in our study, as previously reported [14, 23], because there is a lack of consensus on what reference gene should be used [14, 23]. Reference genes such as U6 and 5S rRNA in the serum or plasma have been used in previous studies; however, they were found to be unstable in other studies [13, 24]. Fabian Benz reported that the levels of U6 were significantly upregulated in the sera of patients with critical illness and sepsis and were correlated with inflammation. In contrast, levels of spiked-in SV40 displayed a significantly higher stability [25]. Therefore, we do not support the use of U6, 5S rRNA or any specific endogenous miRNA as reference genes. As an alternative, we normalized the data to spike-in cel-miRNA-39-3p [14, 23]. Therefore, our reference genes are more stable than those in other studies.

Third, the selection of the control group in our study was better. Compared with the healthy controls in other studies, the control group in our study included patients with COPD, hypertension, diabetes malignant tumour, trauma and so on. There were no significant differences in comorbidities between the sepsis group and the control group, and thus the selection of the control group in our study was more similar to real-world conditions. Distinguishing sepsis patients from healthy controls is easier than distinguishing sepsis patients from patients with infectious diseases and other comorbidities. Therefore, the results of our evaluations are more convincing than those of other studies that used normal healthy controls. There were also several limitations of the study. First, the sample size of the study was small. We only selected 3 patient serum samples in the screening stage and verified the results in 161 patients. The number of patients in the chip screening and verification stage should be greater. Second, the mechanism by which hsa-let-7d-3p regulates sepsis is still unknown. To our knowledge, no functional studies have shown that hsa-let-7d-3p is associated with sepsis. SIGLEC6, THBS2, CD47 and RASSF8, which are predicted target genes of hsa-let-7d-3p by bioinformatics analysis, are associated with inflammation and immunity [26–29]. RASSF8 is associated with the NF-κB pathway [30]. These target genes need to be further studied in vitro or in vivo.

## Conclusion

We revealed for the first time that hsa-let-7d-3p may be a novel biomarker for the early diagnosis of sepsis. Further study is needed to verify the connection and explore the mechanism.

## Supplementary Information

**Additional file 1: Table S1**: The characteristics of the three patients using for mi-RNA chip

**Additional file 2: Table S2**: Clinical characteristics of the 20 sepsis patients

## Data Availability

The datasets used and/or analysed are available from the corresponding author upon reasonable request.

## Abbreviations

miRNA: MicroRNA
RT-qPCR: Real-time quantitative polymerase chain reaction
ROC: Receiver operating characteristic curve
CRP: C-reactive protein
PCT: Procalcitonin
HIV: Human immunodeficiency virus
SOFA: Sequential Organ Failure Assessment Score
SIRS: Systemic inflammatory response syndrome
COPD: Chronic obstructive pulmonary disease
