# Supplementary material for: Evidence for the circulating microRNA hsa-let-7d-3p as a potential new biomarker for sepsis in human subjects
Source: Eur J Med Res. 2022 Jul 30;27:137. doi: 10.1186/s40001-022-00763-3 (PMC9338616; doi:10.1186/s40001-022-00763-3)
Supplement: Supplementary file 1 — Additional file 1: Table S1: The characteristics of the three patients using for mi-RNA chip [file 40001_2022_763_MOESM1_ESM.docx]

| number | Time interval of serum collection | SOFA scors | SOFA scors(condition improved） | PCT,ng/ml  (sepsis) | PCT,ng/ml  (conditionimproved) |
| --- | --- | --- | --- | --- | --- |
| 1 | 37 | 9 | 0 | 6.60 | 0.08 |
| 2 | 11 | 12 | 4 | 35.18 | 0.24 |
| 3 | 10 | 8 | 1 | 12.09 | 0.34 |

Table 1 The characterist of the three patients using for mi-RNA chip
