# Supplementary material for: Evidence for the circulating microRNA hsa-let-7d-3p as a potential new biomarker for sepsis in human subjects
Source: Eur J Med Res. 2022 Jul 30;27:137. doi: 10.1186/s40001-022-00763-3 (PMC9338616; doi:10.1186/s40001-022-00763-3)
Supplement: Supplementary file 2 — Additional file 2: Table S2: Clinical characteristics of the 20 sepsis patients [file 40001_2022_763_MOESM2_ESM.docx]

Table 2 Clinical characteristics of the 20 sepsis patients

| ID | infection sets | Comorbidity | ApacheⅡscore on admission | Sofa score on admmison | Time to condition improved (days) |
| --- | --- | --- | --- | --- | --- |
| 1 | pneumonia | COPD | 13 | 3 | 9 |
| 2 | pneumonia | OP,Femoral Neck Fractures | 14 | 3 | 23 |
| 3 | pneumonia | CTD,ILD,DM | 18 | 4 | 6 |
| 4 | pneumonia | Lung cancer,OP | 15 | 2 | 13 |
| 5 | pneumonia | COPD,Pulmonary Heart Disease | 14 | 5 | 8 |
| 6 | pneumonia | COPD,Hypertension,CHD,CHF | 12 | 5 | 20 |
| 7 | pneumonia | CTD | 18 | 4 | 16 |
| 8 | pneumonia | DM, | 19 | 4 | 19 |
| 9 | pneumonia | Hypertension,Jaundice | 11 | 5 | 23 |
| 10 | pneumonia | Hypothyroidism | 2 | 1 | 11 |
| 11 | pneumonia | CVD | 12 | 4 | 7 |
| 12 | pneumonia,  intra-abdominal infections |  | 20 | 9 | 37 |
| 13 | pneumonia | Hypertension | 10 | 2 | 17 |
| 14 | pneumonia | Hypertension,CHD,CVD, | 16 | 6 | 11 |
| 15 | pneumonia,  intra-abdominal infections | Trauma | 15 | 7 | 11 |
| 16 | pneumonia |  | 15 | 12 | 11 |
| 17 | urosepsis | Hypertension,DM,Urolithiasis | 10 | 8 | 10 |
| 18 | pneumonia | Trauma,Hypertension,CHD | 19 | 8 | 10 |
| 19 | intra-abdominal infections | bile duct carcinoma after stent implantation,Cholelithiasis, | 16 | 5 | 6 |
| 20 | intra-abdominal infections | CRF,ARF | 12 | 9 | 6 |

12,16,17 were selected to screening differentially expressed miRNAs with miRCURY^TM^ LNA Array.

Abbreviations: COPD, chronic obstructive pulmonary disease; CTD, connective tissue disease; ILD, interstitial lung disease; DM, diabetes mellitus; CHD, coronary heart disease; CHF, chronic heart failure; CVD, coronary heart disease.
